# *Ex Vivo* Restimulation of Human PBMC Expands a CD3^+^CD4^−^CD8^−^ *γδ* ^+^ T Cell Population That Can Confound the Evaluation of CD4 and CD8 T Cell Responses to Vaccination

**DOI:** 10.1155/2013/186420

**Published:** 2013-08-26

**Authors:** B. J. Sedgmen, L. Papalia, L. Wang, A. R. Dyson, H. A. McCallum, C. M. Simson, M. J. Pearse, E. Maraskovsky, D. Hung, P. P. Eomois, G. Hartel, M. J. Barnden, S. P. Rockman

**Affiliations:** ^1^R&D Division, CSL Limited, Parkville, VIC 3052, Australia; ^2^Influenza R&D Division, bioCSL Pty., Ltd., 63 Poplar Road, Parkville, VIC 3052, Australia

## Abstract

The measurement of vaccine-induced humoral and CD4^+^ and CD8^+^ cellular immune responses represents an important correlate of vaccine efficacy. Accurate and reliable assays evaluating such responses are therefore critical during the clinical development phase of vaccines. T cells play a pivotal role both in coordinating the adaptive and innate immune responses and as effectors. During the assessment of cell-mediated immunity (CMI) in subjects participating in a large-scale influenza vaccine trial, we identified the expansion of an IFN-*γ*-producing CD3^+^CD4^−^CD8^−^ *γδ* ^+^ T cell population in the peripheral blood of 90/610 (15%) healthy subjects. The appearance of CD3^+^CD4^−^CD8^−^ *γδ* ^+^ T cells in the blood of subjects was transient and found to be independent of the study cohort, vaccine group, subject gender and ethnicity, and *ex vivo* restimulation conditions. Although the function of this population and relevance to vaccination are unclear, their inclusion in the total vaccine-specific T-cell response has the potential to confound data interpretation. It is thus recommended that when evaluating the induction of IFN-*γ*-producing CD4^+^ and CD8^+^ immune responses following vaccination, the CD3^+^CD4^−^CD8^−^ *γδ* ^+^ T cells are either excluded or separately enumerated from the overall frequency determination.

## 1. Introduction

The hallmark of effective cell-mediated immunity (CMI) in response to vaccination is the generation of long-lasting protective immune memory [1]. Although most vaccines are capable of inducing memory responses, the magnitude of these responses may vary depending on the age and health status of the subject, as well as environmental and genetic factors [2].

Vaccination against seasonal influenza promotes the differentiation of B cells and the production of neutralising antibodies that are specific for the strains of virus formulated in the vaccine. The major viral epitopes against which antibodies are induced are the dominant surface glycoproteins, hemagglutinin (HA), and neuraminidase (NA). The anti-HA and anti-NA antibodies act in concert to limit viral replication by preventing virus from infecting or budding cells, respectively, [3]. Antigen processing via the major histocompatibility complex (MHC) Classes I and II pathways and subsequent induction of a CD4^+^ and, to a lesser extent, CD8^+^ T cell (CTL) responses are also observed following vaccination with seasonal inactivated influenza vaccines [4]. Influenza virus-specific CTLs provide broad protection against influenza virus infection. This is a consequence of granule-mediated cytotoxicity and the production of IFN-*γ* and other cytokines, which together limit viral replication, and consequently reduce infection-associated mortality and morbidity [1, 5–7].

Following antigen exposure, naïve *αβ* expressing T cells (CD4^+^ and CD8^+^) are activated and subsequently proliferate and differentiate into effector cells. Over time, the effector T cell populations contract and give rise to small populations of antigen-specific memory T cells [8, 9]. There is also another subset of T cells expressing the *γδ* T cell receptor (*γδ* TCR) that recognises and processes foreign antigen [10, 11]. These cells represent approximately 1–10% of human peripheral blood T cells. Unlike *αβ* T cells, *γδ* ^+^ T cells recognise distinct antigens and display distinct kinetics with unique functional potential at specific anatomical sites (reviewed in Vantourant and Hayday, 2013). Although the effector function of *γδ* ^+^ T cells has been widely observed in a variety of bacterial, viral, and protozoan infections, their specific role(s) in immune surveillance and protection are not well understood. 

In the present study, a saponin-based adjuvant (ISCOMATRIX adjuvant) developed by CSL Limited was formulated with influenza antigens and tested in four study cohorts of healthy adult subjects which differed on the basis of age and disease susceptibility (i.e., young adults versus elderly adults living in or out of long-term care facilities). One of the aims of this study was to investigate the capacity of the test vaccines to induce CD4^+^ and CD8^+^ T cell responses in the different study cohorts, as these cells are known to be important in providing protection against influenza infection. During the evaluation of the vaccine-induced T cell responses, we also detected a *γδ* ^+^ T cell population in a proportion (~15%) of subjects following the culture of peripheral blood mononuclear cells (PBMC) in the presence of influenza antigens. The presence of this population was independent of study cohort, vaccine group, subject gender, and ethnicity or *ex vivo* stimulation conditions. The function of this population and relevance to vaccination are unclear, and their inclusion in the total vaccine-specific T cell responses has the potential to confound data interpretation. It is thus recommended that when evaluating the induction of IFN-*γ*-producing CD4^+^ and CD8^+^ immune responses following vaccination, the CD3^+^CD4^−^CD8^−^ *γδ* ^+^ T cells are either excluded or separately enumerated from the overall frequency determination. 

## 2. Materials and Methods

### 2.1. Study Design

Four study cohorts were enrolled to examine the immunogenicity of three formulations of a trivalent-split-virion-influenza-ISCOMATRIX vaccine and a licensed seasonal influenza vaccine in a prospective, randomised double-blind, multicentre trial (Phase II trial, ClinicalTrials.gov identifier: NCT00479648). The cohorts were adults aged ≥18 to ≤45 years (Cohort A 18–45); adults aged ≥60 to <75 yrs (Cohort B 60–74); adults aged ≥75 yrs (Cohort C 75^+^); and adults aged ≥60 yrs who were residents of long-term care facilities (Cohort D 60^+^ LTCF).

### 2.2. Vaccines and Viruses

The investigational study vaccines delivered to subjects within the study cohorts contained 15 *μ*g of influenza antigen from each of the three influenza virus strains: A/New Caledonia/20/99 (H1N1); A/Hiroshima/52/2005 (H3N2); and B/Malaysia/2506/2004 either alone or combined with 22, 45, or 90 ISCO units of ISCOMATRIX adjuvant per 0.5 mL dose. The seasonal influenza vaccine was a European licensed, trivalent, inactivated, split-virus vaccine (with the same strains as aforementioned) prepared in embryonated hen eggs, using standard techniques by bioCSL (Parkville, Australia). Essentially the same manufacturing methods that were used to prepare the virus strains used in the study vaccines.

### 2.3. Vaccination Regime and Whole Blood Collection

Following collection of a prevaccination bleed (day 0), enrolled subjects received a single dose of 0.5 mL of study vaccine by intramuscular injection. Whole blood was then collected from subjects on days 7 and 30 after vaccination for the preparation of PBMCs.

### 2.4. PBMC Preparation and *Ex Vivo* Intracellular Cytokine Assay

PBMCs were isolated from whole blood by Ficoll-Paque Plus (GE Healthcare Bio-Sciences AB, Uppsala, Sweden) using density gradient separation and cryopreserved in liquid nitrogen in 90% foetal bovine serum (FBS; SAFC Biosciences, KS, USA) and 10% DMSO until analyzed. 

An intracellular cytokine (IFN-*γ*) assay was performed using PBMCs from all time points (0, 7, and 30 days) for a given subject to examine their antigen-specific CD4^+^ and CD8^+^ T cell responses to influenza vaccination. Briefly, PBMCs (1 × 10^6^ /well) were thawed and cultured for 16 hours (37°C, 5% CO_2_) in RP10 (RPMI-1640 containing 10% FBS, 1 mM GlutaMAX-I supplement, 55 *μ*M 2-mercaptoethanol, 50 U/mL penicillin, 50 *μ*g/mL streptomycin, and 10 mM HEPES) with anti-CD28 and anti-CD49d (1 *μ*g/mL each; both from BD Biosciences, CA, USA) and influenza antigen in either the form of pooled virus vaccine strains (30 HA units per strain/mL) or CTL epitopes (3 *μ*g/mL). Brefeldin A (BFA; 5 *μ*g/mL; Sigma-Aldrich Co., MO, USA) was included for the final 14 hours. The PBMCs were then fixed, permeabilised, and labelled with cell-surface-marker-specific antibodies (anti-CD3 FITC, anti-CD4 PerCP Cy5.5, and anti-CD8 APC; all from BD Biosciences) and anti-IFN-*γ* PE (Invitrogen Corporation, CA, USA) to detect the intracellular cytokine. 

The unexpected IFN-*γ*-producing CD3^+^CD4^−^CD8^−^ T-cell population observed in some of the subjects enrolled in this study was further immunophenotyped. Using the method described previously, restimulated PBMCs were stained with anti-TCR *αβ* FITC, anti-V*α*24 FITC, and anti-CD161 FITC (kindly provided by Professor D. Godfrey and Associate Professor S. Berzins) to confirm the identity of the population as being *γδ* ^+^ T cells. The induction of IFN-*γ* production from these *γδ* ^+^ T cells was then able to be assessed by staining with anti-CD3 FITC, anti-CD4 PerCP Cy5.5 (or anti-CD8 PerCP Cy5.5), and anti-TCR *γδ* APC (all from BD Biosciences), as well as anti-IFN-*γ* PE. 

### 2.5. Data Analysis

Stained/fixed PBMCs were acquired on a FACSCalibur (Becton Dickinson) and antigen-specific CD4^+^ and CD8^+^ T cells analysed using FlowJo software (Tree Star Inc., OR, USA). Typically 300,000 cellular events from each sample were captured through a lymphocyte gate. FlowJo software was also used for post acquisition analysis of data to retrospectively exclude IFN-*γ*-producing *γδ* ^+^ T cell populations from the final dataset. Chi-squared tests by the statistical program SAS Enterprise Guide 4.3 were used to compare subjects according to study cohort, vaccine group, and subject gender and ethnicity.

## 3. Results

In the present study, we evaluated CMI responses in 610 healthy adult subjects who were administered a seasonal influenza vaccine either alone or formulated with ISCOMATRIX adjuvant. Subjects were allocated to one of four cohorts that included young adults and elderly adults who were either living in the community or residents of long-term care facilities. In 15% of enrolled subjects (90/610), an unexpected IFN-*γ*-producing *γδ* ^+^ T cell population which expressed CD3 but neither CD4 nor CD8 surface antigens was identified by flow cytometric analysis following *ex vivo* restimulation of PBMC. The frequencies at which this population was detected in each of the four cohorts were not significantly different from one another (*P* = 0.85) (Table 1). 

The presence of this IFN-*γ*-producing CD3^+^CD4^−^CD8^−^ *γδ* ^+^ T cell population was found to be independent of vaccine formulation and sample time point and, in the majority of subjects, appeared transiently with most “responders” showing a detectable population at only one of the three time points analysed (Table 2). It was observed from this analysis that subjects aged 60 years and older (i.e., Cohorts B–D) contained a higher number of “responders” at day 7 when compared to the younger subjects in Cohort A, although this was not significant (*P* = 0.16). Furthermore, the number and percentage of *γδ* ^+^ T cell responders within Cohort A were significantly higher at day 30 after vaccination (*P* < 0.01) suggesting that, although the overall frequency of “responders” is not significantly different between study cohorts, there may be an age-related effect in the timing of *γδ* ^+^ T cell induction within a study cohort. Subject gender and ethnicity were found not to correlate with the presence of this CD3^+^CD4^−^CD8^−^ *γδ* ^+^ T cell population (data not shown).

**Figure 1:**
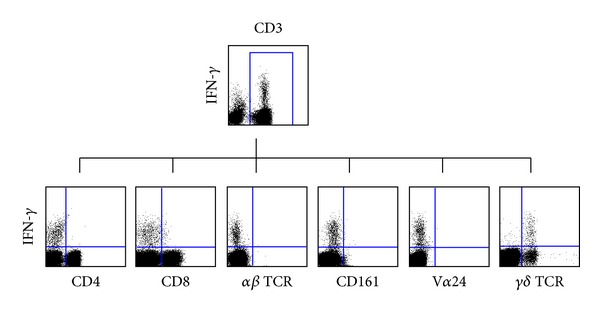
Characterisation of an IFN-*γ*-producing CD3^+^CD4^−^CD8^−^ *γδ* ^+^ T cell-positive subject at day 7 after vaccination. PBMCs were expanded *in vitro* in the absence of exogenous influenza virus antigen, and the expression of CD4, CD8, *αβ* TCR, CD161, V*α*24, and *γδ* TCR surface antigens was assessed by flow cytometry.

A panel of monoclonal antibodies (mAbs) to cell surface antigens were used to immunophenotype the CD3^+^CD4^−^CD8^−^ *γδ* ^+^ T cell population (Figure 1). Lack of reactivity of this cell population with both anti-V*α*24 and anti-CD161 antibodies effectively ruled them out as being IFN*γ*-producing natural killer T (NKT) cells. However, these cells did stain positively with an anti-*γδ* monoclonal antibody suggesting that the CD3^+^CD4^−^CD8^−^ IFN-*γ*-producing T cell population consists of *γδ* T cells. 

The CD3^+^CD4^−^CD8^−^ *γδ* ^+^ IFN-*γ*-producing T cell population was further characterised to elucidate the factors that may promote their expansion *ex vivo*. The expansion of these cells was not observed immediately after thawing fresh PBMCs, but rather they emerged in the culture (data not shown). Furthermore, the expansion of this population was shown to be independent of exogenous influenza antigen stimulation, as they were also observed following overnight culture in the absence of influenza antigen (data not shown).

To investigate the possibility that the observed CD3^+^CD4^−^CD8^−^ *γδ* ^+^ IFN-*γ*-producing T cell population was the result of an unexpected staining artefact, an additional washing step was introduced to exhaustively remove unbound, excess fluorescently labelled antibodies prior to flow cytometric analysis. This additional step had no effect on both the number of subjects who had this population and the magnitude of the population, suggesting that this cell population was “real” and not a consequence of a staining artefact (data not shown). 

**Figure 2:**
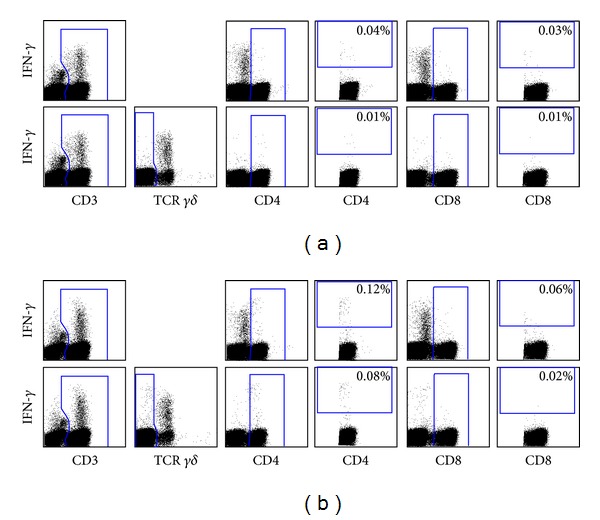
Alternative gating strategy applied to influenza vaccine trial data to eliminate confounding CMI responses from the IFN-*γ*-producing *γδ* ^+^ T cells. PBMCs from an IFN-*γ*-producing CD3^+^CD4^−^CD8^−^ *γδ* ^+^ T cell-positive subject at day 7 after vaccination were restimulated in (a) the absence of antigen or (b) the presence of exogenous influenza virus antigen (30 HA units per strain/mL), and antigen-specific CD4^+^ and CD8^+^ T-cell responses were assessed. For each of the restimulation conditions, the top panels represent the standard gating strategy, and the bottom panels represent the alternative gating strategy, whereby CD3^+^ *γδ* ^+^ T cells were selectively excluded during post acquisition analysis to avoid reporting misleading cytokine responses that could confound the antigen-specific CD4^+^ and CD8^+^ T cell responses.

In order to exclude the contribution of the CD3^+^CD4^−^CD8^−^ *γδ* ^+^ IFN-*γ*-producing T cell population so as to more accurately enumerate vaccine-specific CD4^+^ and CD8^+^ cellular responses, an alternate gating strategy which eliminated this population was adopted (Figure 2).

## 4. Discussion

The present study reports, for the first time, the transient appearance of an IFN-*γ*-producing CD3^+^CD4^−^CD8^−^ *γδ* ^+^ T-cell population following restimulation of PBMCs collected from subjects after receiving an influenza vaccine. Further characterisation of this population, which was detected in 90/610 (15%) enrolled subjects, revealed that the induction of the *γδ* ^+^ T cell response was unrelated to the age of subjects, vaccine group, and *ex vivo* restimulation conditions including exogenous influenza antigen and costimulation. Furthermore, the presence of this population was found to be independent of subject gender and ethnicity. 

The correlation between subject age and normal reference ranges of *γδ* ^+^ T cells in healthy subjects has previously been published by Andreu-Ballester et al. (2012). It was shown that the absolute number of CD3^+^CD4^−^CD8^−^ *γδ* ^+^ T cells was significantly lower in subjects aged 71 years and above [12] compared with younger subjects. Although the absolute numbers of CD3^+^CD4^−^CD8^−^ *γδ* ^+^ T cells and other *γδ* ^+^ T-cell subsets were not reported in the present study, it would be of interest to further investigate the relative effector responses of each of these subsets at various time points after vaccination in large-scale vaccination trials.

We propose, although not formally proven, that the *γδ* ^+^ T cells detected in PBMCs after restimulation may have arisen as a consequence of the expansion of a “memory pool” of *γδ* ^+^ T cells generated in response to a previous unrelated bacterial, viral, or parasitic protozoan infection. “Innate-like” activation of circulating *γδ* ^+^ T cells in humans is mediated following recognition of an activating ligand (e.g., the phosphoantigen, isopentenyl pyrophosphate (IPP)) presented by an unidentified surface molecule on an antigen-presenting cell or cells (APC) [13]. Polyclonal expansion of effector *γδ* ^+^ T cells has been shown to occur in a wide range of disease models including tuberculosis, malaria, Leishmaniasis, HIV, and tularaemia [14–19]; and this response correlates with enhanced regulation of pathogen clearance, inflammation, and wound repair [20]. More recently it has been suggested by Qin et al. (2011) that phosphoantigen IPP-expanded human *γδ* ^+^ T cells have a higher capacity to produce IFN-*γ* and exhibit cytolytic and noncytolytic effects against influenza-infected cells [21]. However, the induction of IFN-*γ*-producing CD3^+^CD4^−^CD8^−^ *γδ* ^+^ T cells in the present study was found not to be related to influenza antigen. A possible reason for the discrepancy between our findings and those of Qin et al., could stem from the deliberate IPP stimulation of *γδ* ^+^ T cells to promote their effector function against influenza-infected cells. Instead, it is reasonable to hypothesise that a proportion of subjects (i.e., 15%) may have had a preexisting infection or autoimmune disease which resulted in the exposure of IPP or another activating ligand which, inturn, may have primed the transient antigen-independent expansion of effector *γδ* ^+^ T cells following *ex vivo *restimulation of PBMC. The production of IFN-*γ* from these cells collected before and after vaccination occurred after several hours in culture, but the exact trigger of this effector response is yet to be determined.

In addition to their defensive role in microbial infections, human *γδ* ^+^ T cells are also known to be cytotoxic against some tumours following the recognition of stress-associated antigens. The overproduction of IPP by transformed cells has been shown to result in the activation and effector function of *γδ* ^+^ T cells, most notably by the immediate release of cytokines such as IFN-*γ* and TNF-*α* [22, 23]. 

Recently it has been shown that psychological stress mobilises *γδ* ^+^ T cells into peripheral blood in much the same way as has been previously reported for cytotoxic CD8 and NK cells. Furthermore, these mobilised *γδ* ^+^ T cells have also been found to be highly cytotoxic through their ability to rapidly recognize their targets in an “innate-like” manner [24]. With this in mind, an alternate explanation for the emergence of this population in PBMC cultures during our study may relate to psychological stress in 15% of subjects at the time of vaccination and/or blood draw. The correlation of stress and immune cell mobilisation has previously been linked to enhanced immunologic memory in a murine model, whereby local lymphocyte and macrophage numbers as well as type 1 cytokines including IFN-*γ* were shown to be enhanced after vaccination [25]. The kinetics of *γδ* ^+^ T cell proliferation, mobilisation, and IFN-*γ* production in psychologically stressed humans remains unknown; however, it is tempting to speculate that this is a transient effector response. This would therefore explain why the expansion of IFN-*γ*-producing *γδ* ^+^ T cells in PBMC cultures was mostly observed in only one of the three time-points analysed. 

We have reported the presence of an IFN-*γ*-producing CD3^+^CD4^−^CD8^−^ *γδ* ^+^ T cell population in a large-scale influenza vaccination trial which is unrelated to the study cohort, vaccine group, the gender and ethnicity of subjects, and the *ex vivo* re-stimulation conditions of their PBMCs. Although we have not yet determined the mechanism attributable for their expansion *ex vivo*, the presence of IFN-*γ*-producing CD3^+^CD4^−^CD8^−^ *γδ* ^+^ T cells has the potential to give overestimated T cell responses in large-scale vaccination trials involving influenza or other antigens when only effector CD4^+^ and CD8^+^ immune responses are required. It is therefore recommended that when evaluating the induction of IFN-*γ*-producing CD4^+^ and CD8^+^ immune responses following vaccination, the CD3^+^CD4^−^CD8^−^ *γδ* ^+^ T cells are either excluded or separately enumerated from the overall frequency determination. 

## Figures and Tables

**Table 1:** Number of subjects and percentage of IFN-*γ*-producing CD3^+^CD4^−^CD8^−^ *γδ* ^+^ T-cell responders and nonresponders detected in each of the four study cohorts which comprised of various formulations of influenza and ISCOMATRIX adjuvant. The number of CD3^+^CD4^−^CD8^−^ *γδ* ^+^ T-cell responders was compared between cohorts by a Chi-squared test using the statistical program SAS Enterprise Guide 4.3.

Cohort	No. of subjects	*γδ* ^ +^ T-cell responders		*γδ* ^ +^ T-cell nonresponders	
		*N*	%	*N*	%
A (≥18 to ≤45 years)	186	25*	13.4	161	86.6
B (≥60 to <75 years)	240	36*	15.0	204	85.0
C (≥75 years)	79	11*	13.9	68	86.1
D (≥60 years in LTCF)	105	18*	17.1	87	82.9

All	610	90	14.8	520	85.2

*P = 0.85.

**Table 2:** Number of subjects and percentage of IFN-*γ*-producing CD3^+^CD4^−^CD8^−^ *γδ* ^+^ T cell responders only detected at a single time point (i.e., day 0, day 7, or day 30) or multiple time points within each of the four study cohorts which were comprised of various formulations of influenza and ISCOMATRIX adjuvant. The number of CD3^+^CD4^−^CD8^−^ *γδ* ^+^ T cell responders was compared between cohorts by a Chi-squared test using the statistical program SAS Enterprise Guide 4.3.

*γδ* ^ +^ T cell responders	Cohort A (≥18 to ≤45 years)		Cohort B (≥60 to <75 years)		Cohort C (≥75 years)		Cohort D (≥60 years in LTCF)	
	*N*	%	*N*	%	*N*	%	*N*	%
Day 0 only	4	16.0	8	22.2	2	18.2	4	22.2
Day 7 only	7	28.0	21	58.3	9	81.8	9	50.0
Day 30 only	10	40.0	6	16.7	0	0.0	1	5.6
Days 0 and 7	0	0.0	0	0.0	0	0.0	2	11.1
Days 0 and 30	1	4.0	0	0.0	0	0.0	0	0.0
Days 7 and 30	3	12.0	0	0.0	0	0.0	1	5.6
Days 0, 7, and 30	0	0.0	1	2.8	0	0.0	1	5.6

Any day	25*	100.0	36*	100.0	11*	100.0	18*	100.0

*P = 0.85.
